# Noninvasive positive pressure ventilation enhances the effects of aerobic training on cardiopulmonary function

**DOI:** 10.1371/journal.pone.0178003

**Published:** 2017-05-22

**Authors:** Takashi Moriki, Takeshi Nakamura, Yoshi-ichiro Kamijo, Yukihide Nishimura, Motohiko Banno, Tokio Kinoshita, Hiroyasu Uenishi, Fumihiro Tajima

**Affiliations:** 1 Department of Rehabilitation Medicine, Wakayama Medical University, Wakayama city, Wakayama, Japan; 2 Department of Rehabilitation Medicine, Yokohama City University, School of Medicine, Yokohama city, Kanagawa, Japan; Kurume University School of Medicine, JAPAN

## Abstract

**Purpose:**

The purpose of this study was to determine the effect of aerobic training under noninvasive positive pressure ventilation (NPPV) on maximal oxygen uptake ($\dot{V}O_{2\max}$).

**Methods:**

Ten healthy young male volunteers participated in the study. Before the training, stroke volume (SV) and cardiac output (CO) were measured in all subjects under 0, 4, 8, and 12 cmH_2_O NPPV at rest. Then, the subjects exercised on a cycle ergometer at 60% of pre-training $\dot{V}O_{2\max}$ for 30 min daily for 5 consecutive days with/without NPPV. The 5-day exercise protocol was repeated after a three-week washout period without/with NPPV. The primary endpoint was changes in $\dot{V}O_{2\max}$. The secondary endpoints were changes in SV, CO, maximum heart rate (HR_max_), maximum respiratory rate (RR_max_), maximum expiratory minute volume (VE_max_) and the percent change in plasma volume (PV).

**Results:**

NPPV at 12 cmH_2_O significantly reduced SV and CO at rest. $\dot{V}O_{2\max}$ significantly increased after 5 days training with and without NPPV, but the magnitude of increase in $\dot{V}O_{2\max}$ after training under 12 cmH_2_O NPPV was significantly higher than after training without NPPV. VE_max_ significantly increased after training under NPPV, but not after training without NPPV. HR_max_ and RR_max_ did not change during training irrespective of NPPV. The percent change in PV was similar between training with and without NPPV. The 5-day training program with NPPV resulted in greater improvement in $\dot{V}O_{2\max}$ than without NPPV.

**Conclusions:**

Aerobic training under NPPV has add-on effects on $\dot{V}O_{2\max}$ and exercise-related health benefits in healthy young men.

## Introduction

Good cardiopulmonary function is associated with health benefits; a lower risk of all-cause mortality [1–3] and a higher physical work capacity[4,5]. Cardiopulmonary function is often tested to assess fitness, development and appraisal of exercise and training programs. Thus, assessment of cardiopulmonary function is of interest to researchers and clinicians alike. Maximal oxygen uptake ($\dot{V}O_{2\max}$) is considered the criterion measure of cardiopulmonary function [6]. First established by Hill and Lupton [7], $\dot{V}O_{2\max}$ represents the integrated response of the cardiovascular, respiratory and muscular systems to take up, distribute and utilize oxygen during exercise to volitional exhaustion and is one of the most widely used diagnostic tests for both athletic and clinical population groups [8–10]. Estimates of $\dot{V}O_{2\max}$ obtained using maximal exercise protocols are typically based on a performance measure such as time or distance covered [5,11–13] or in cycle ergometer and peak work rate [14].

The $\dot{V}O_{2\max}$ is reduced by prolonged bed rest. Saltin et al. [15] reported that $\dot{V}O_{2\max}$ in 5 healthy 20-years-old men was reduced by an average of 28% after three weeks of bed rest. Long-term bed rest conditions increase the stroke volume (SV) and cardiac output (CO) due to increased venous return from the lower body. The persistent increase in SV and CO induces a decrease in plasma volume (PV) and causes cardiac atrophy, with subsequent fall in $\dot{V}O_{2\max}$ [16]. The circulatory condition induced by application of negative pressure to the lower parts of the body while in supine position, mimics the fall in venous return during upright posture with low SV and CO associated with the effects of gravity. Watenpaugh et al. [17] demonstrated that daily supine lower body negative pressure (LBNP) treadmill exercise at 41–65% of $\dot{V}O_{2\max}$ during 15 days of bed rest can preserve peak $\dot{V}O_{2}$ at pre-bed rest levels.

Noninvasive positive pressure ventilation (NPPV) is a non-invasive treatment used for patients with sleep apnea syndrome and chronic obstructive pulmonary disease [18–20]. NPPV is delivered through a nose/full-face mask instead of endotracheal intubation. In addition to its effect on the respiratory system, NPPV also alters the cardiovascular-circulatory system and including falls in SV and CO [21].

Based on the above background, we hypothesized that aerobic training under NPPV improves the cardiopulmonary function, compared with aerobic training alone. The primary outcome of the present study was changes in $\dot{V}O_{2\max}$. The purpose of this study was to determine the effects of NPPV under rest conditions on circulatory status, and the effects of the combination of NPPV and aerobic training on cardiopulmonary function, including $\dot{V}O_{2\max}$.

## Methods

Ten healthy young men (BMI: 18.1–27.7 kg/m^2^, age: 24–34 years) were recruited from the medical staff of Wakayama Medical University Hospital, Wakayama city, Japan (study period: 01.06.2013–30.10.2013). All subjects completed the study and none dropped-out. Each participant provided a signed informed consent before the commencement of the study. Table 1 summarizes the characteristics of the subjects. All subjects were normotensive, not on medications, and free of cardiovascular or neuromuscular diseases, based on medical history and physical examination. All subjects were active in recreational sports/exercise. The study was approved by the Human Investigation Committee of the Wakayama Medical University, Japan.

**Table 1:** Baseline data.

Characteristics	Total (*n* = 10)
Age (years)	28.3±3.1
Height (cm)	172.3±5.9
Weight (kg)	68.1±12.5
Body Mass Index (kg/m^2^)	22.8±3.0
Resting heart rate (bpm)	65.9±7.3
Resting systolic blood pressure (mmHg)	116.3±7.1
Resting diastolic blood pressure (mmHg)	65.4±8.6
Resting mean blood pressure (mmHg)	82.4±7.6

Data are mean ± SD.

### Assessment of cardiovascular responses to positive pressure ventilation at resting conditions

One week before the start of aerobic training, each subject underwent measurements of SV, CO, arterial blood pressure (BP) and heart rate (HR) during NPPV at pressure levels of 0, 4, 8, 12 cmH_2_O in the supine position. The above parameters were recorded 5 minutes after steady-state breathing at the selected NPPV level. Artificial ventilation (Respironics V60; Philips, Amsterdam, Holland) was used in all subjects with a nasal mask, and the ventilator mode was set to continues positive airway pressure (CPAP) throughout the study. CO and SV were measured by the impedance method (Noninvasive Continuous Cardiac Output Monitor MCO-101; Medisens, Tokyo). HR was obtained from the R-R interval of the electrocardiogram (Stress Test System ML-9000; Fukuda Denshi, Tokyo) and BP was measured manually by a sphygmomanometer.

### Aerobic training

In this cross-over design study, aerobic exercise was performed with and without NPPV (mode; CPAP, at inspired oxygen concentration [FiO_2_] of 21%). Subjects performed on a cycle ergometer exercise in an upright position at 60% of pre-training $\dot{V}O_{2\max}$ for 30 min daily for 5 consecutive days either with or without NPPV. The exercise protocol was repeated with the alternate combination of NPPV after a three-week washout period. NPPV was used in random order among the participating subjects. The exercise was performed at 1700–1900 in an air-conditioned room with the temperature set at 28°C. HR was continuously monitored during the exercise. BP and Borg Scale were measured before and at the end of the exercise on the first day of training with and without NPPV. Subjects were not allowed to drink any fluid during exercise.

### Measured variables

$\dot{V}O_{2\max}$, maximum heart rate (HR_max_), maximum respiratory rate (RR_max_) and maximum expiratory minute volume (VE_max_) were measured 24 hour before the first training (baseline) and 24 hour after last training (post-training). $\dot{V}O_{2\max}$, HR_max_, RR_max_ and VE_max_ were measured with graded exercise using a cycle ergometer in an upright position. $\dot{V}O_{2}$, HR, RR and VE were monitored continuously by expiration gas analyzer (Aeromonitor AE300S; Minato, Tokyo). After baseline measurements at rest for 3 min, the subject started pedaling at 60 cycles/min without load. The exercise intensity was increased by 50 W every 3 min to 150 W and, higher than this intensity, by 20 W every 1 min until exhaustion. $\dot{V}O_{2\max}$, HR_max_, RR_max_ and VE_max_ were determined by averaging the three largest consecutive values at the end of exercise. The ergometer seat and handlebar heights were recorded for each individual subject during the baseline measurements and were used during the post-training measurements.

Blood samples were collected at baseline and post-training from the antecubital vein using heparinized tubes, to measure hemoglobin and hematocrit. The percent change in PV was calculated from the hematocrit and hemoglobin concentrations using the following equation: ΔPV (%) = 100 × (Hb_post_/Hb_C_) × {[1 – (Hct_C_/100)]/[1 – (Hct_post_/100)]} − 100, where ΔPV is the percent change in PV, Hb_C_ is baseline hemoglobin concentration, Hb_post_ is post-training hemoglobin concentration, Hct_C_ is baseline hematocrit, and Hct_post_ is post-training hematocrit [22].

### Statistical analysis

Differences in SV, CO, BP and HR during different NPPV values recorded in supine position were analyzed by one-way repeated measures analysis of variance followed by Tukey-Kramer’s test. The Student’s paired t-test was used to examine for differences between before and after exercise, pre- and post-training, and training under NPPV and without NPPV for each parameter. Data were expressed as mean±SD. A *P* value <0.05 was considered statistically significant. All statistical analyses were performed using statistical analysis software (Graph Pad Prism 6). We calculated the statistical power and the appropriate sample size to detect significant differences that need to be observed in this study. The statistical power was 61.6%, and the necessary sample size was 10 samples.

## Results

### Cardiovascular responses during NPPV at rest

**Fig 1:**
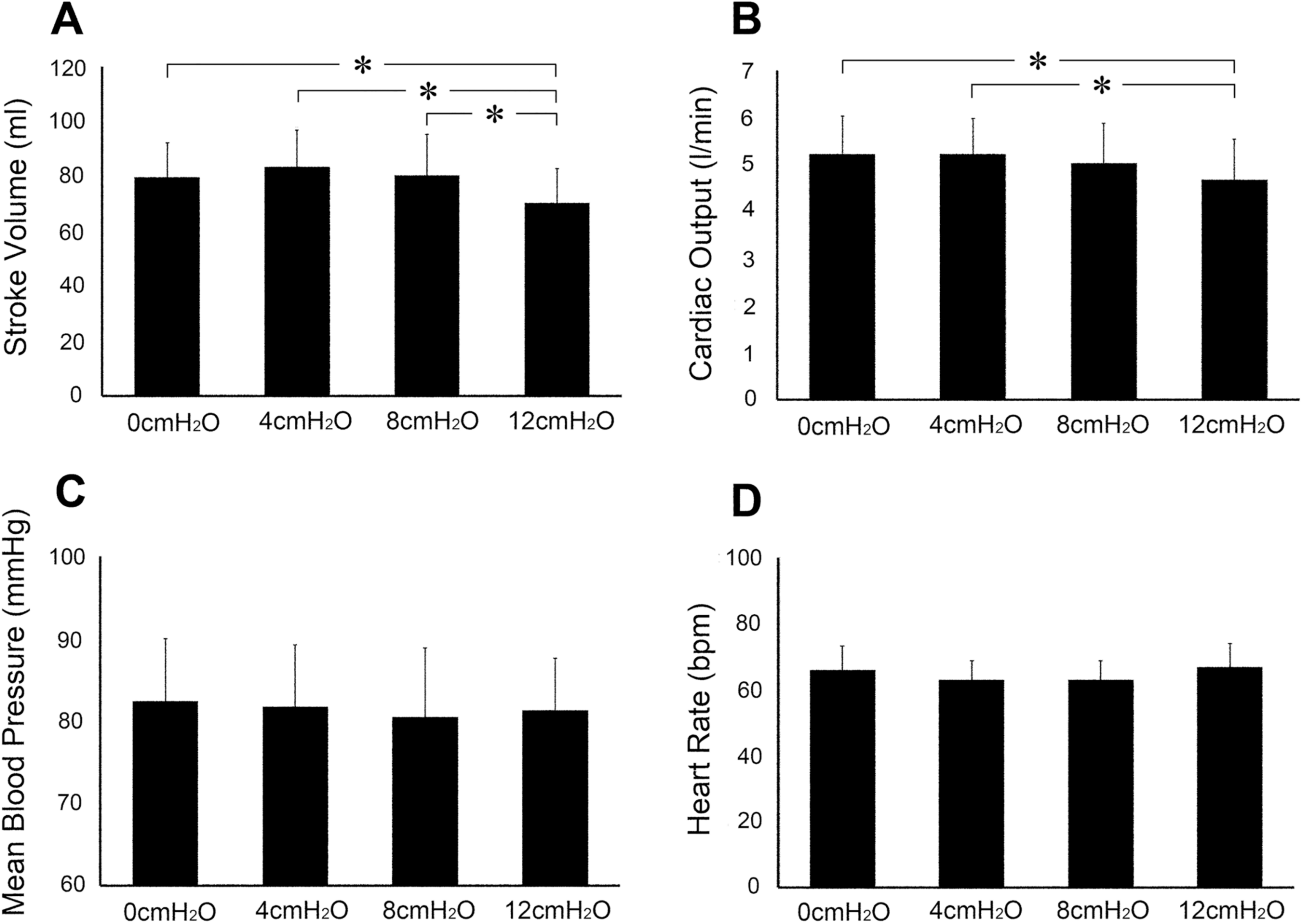
**(A)** stroke volume, **(B)** cardiac output, **(C)** arterial blood pressure and **(D)** heart rate measured in supine position during noninvasive positive pressure ventilation at pressure levels of 0, 4, 8, 12 cmH_2_O. Data are mean±SD. *P < 0.05.

The SV during NPPV of 12 cm H_2_O was significantly lower (70.4±12.4 ml) than at pressure level of 0, 4, and 8 cm H_2_O (79.7±12.5, 83.4±13.5, and 80.4±14.9 ml, respectively, P<0.05) (Fig 1A). The CO during NPPV of 12 cm H_2_O (4.7±0.9 l/min) was significantly lower (P<0.05) than at NPPV of 0 and 4 cm H_2_O (5.2±0.8 and 5.2±0.8 l/min, respectively), but not at 8 cm H_2_O (5.0±0.9 l/min) (Fig 1B). NPPV had no effect on the mean blood pressure (MBP) (0, 4, 8 and 12 cm H_2_O: 82.4±7.6, 81.7±7.6, 80.4±8.5 and 81.3±6.4 mmHg, respectively) and HR (0, 4, 8 and 12 cm H_2_O: 65.9±7.3, 62.8±6.0, 62.9±5.8 and 66.7±7.2 bpm, respectively) (Fig 1C and 1D). Based on these findings, NPPV of 12 cmH_2_O was used during aerobic training.

### Effects of NPPV on HR, MBP and Borg scale during exercise

**Fig 2:**
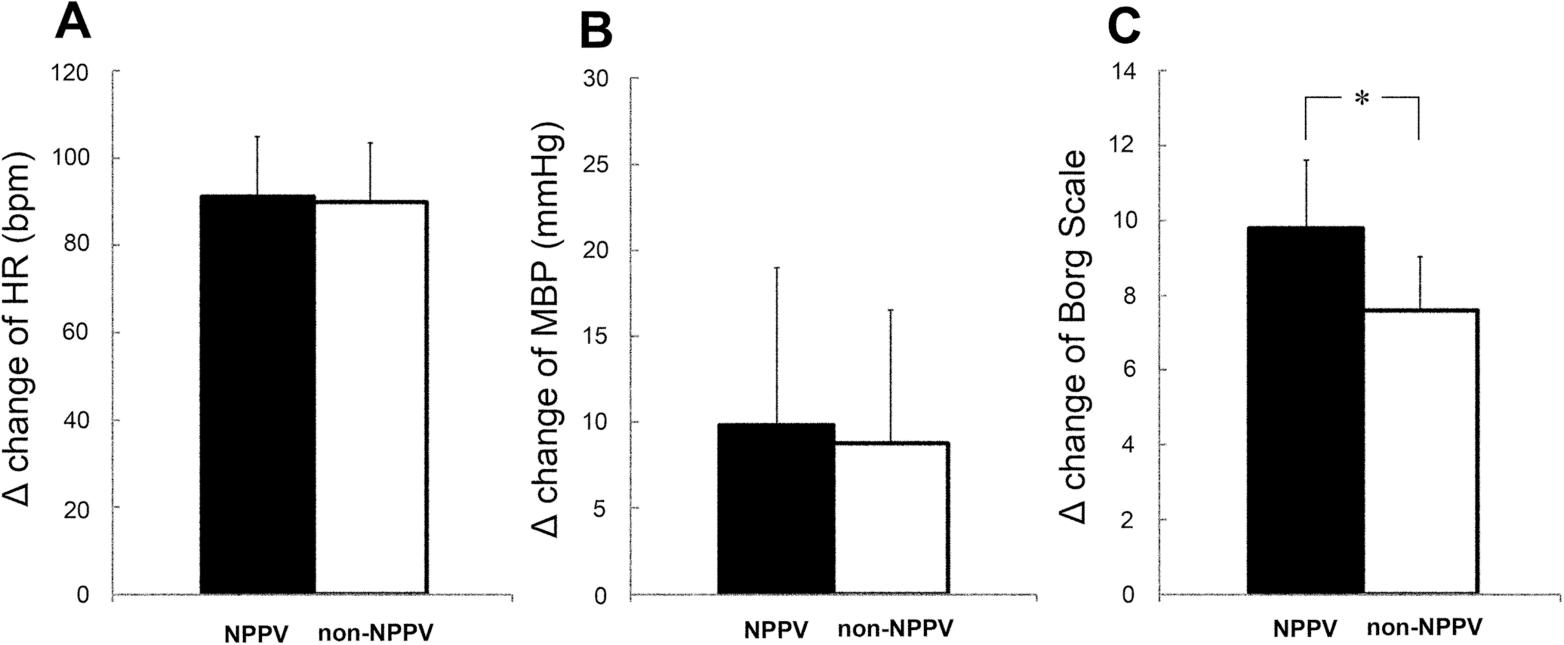
Delta changes in **(A)** heart rate (HR), **(B)** mean blood pressure (MBP), and **(C)** Borg scale during exercise on the first day of training with and without NPPV (NPPV and non-NPPV). Data are mean±SD. *P<0.05.

At baseline with the subject in sitting position, NPPV had no effect on MBP and Borg Scale (MBP: control: 84.2±5.4, NPPV: 83.7 mmHg, Borg scale: control: 8.0±0.8, NPPV: 7.6±0.7), but it significantly increased HR (control: 75.1±6.1, NPPV: 80.4±7.5 bpm, P<0.05). All three variables increased significantly (P<0.05) during exercise. However, NPPV had no effect on HR and MBP during exercise (HR: no NPPV: 90.0±13.4, NPPV: 91.1±14.0 bpm, MBP: no NPPV: 8.8±7.7, NPPV: 9.8±9.2 mmHg) (Fig 2A and 2B). However, NPPV significantly increased the Borg Scale during exercise (no NPPV: 7.6±1.4, NPPV: 9.8±1.8, P<0.05) (Fig 2C).

### Effects of NPPV on $\dot{V}O_{2\max}$, HR_max_, VE_max_ and RR_max_

**Fig 3:**
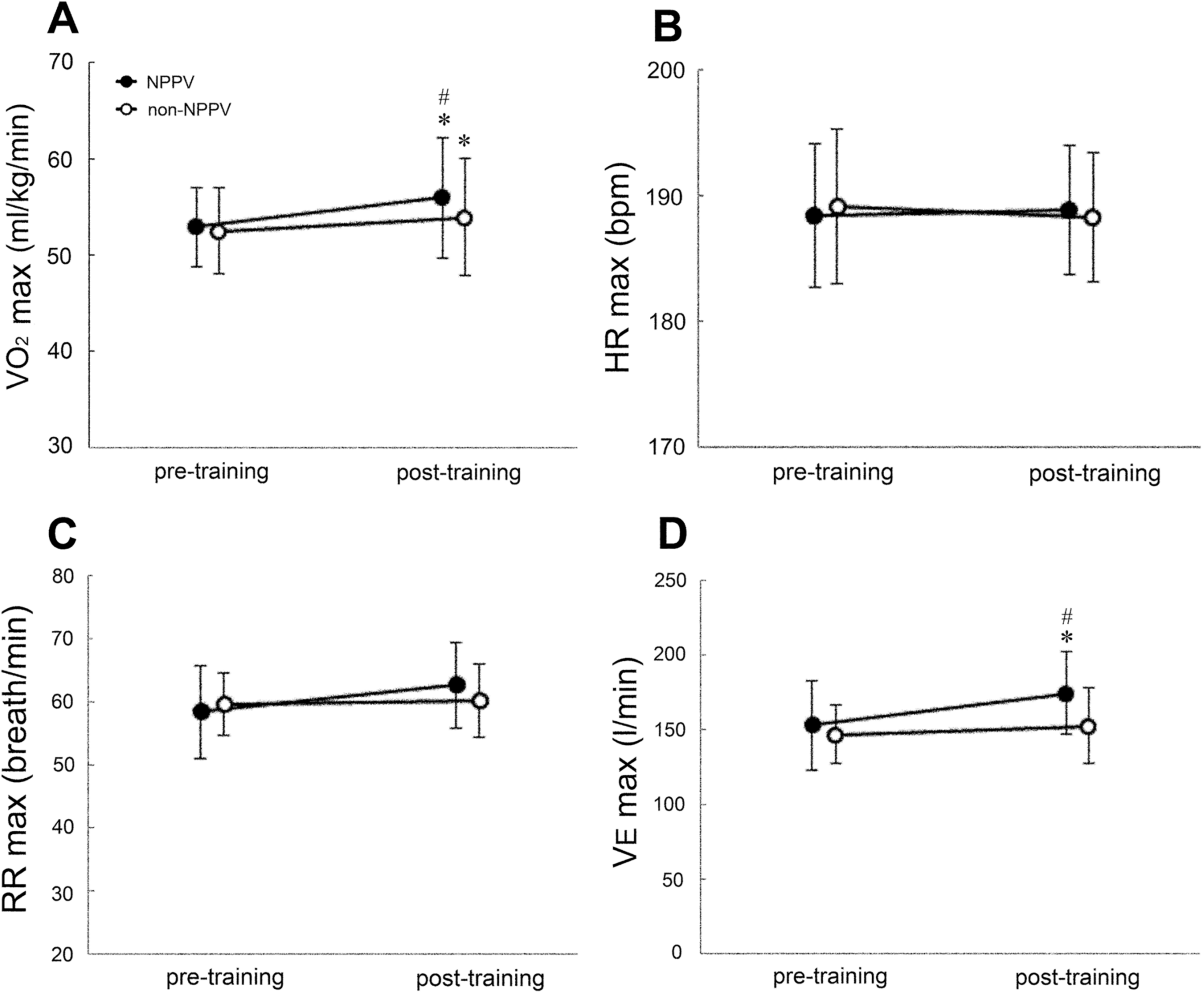
**(A)** Maximal oxygen uptake ($\dot{V}O_{2\max}$), **(B)** maximum heart rate (HR_max_), **(C)** maximum respiratory rate (RR_max_), and **(D)** maximum expiratory minute volume (VE_max_) at 24 hour before the first training (pre-training) and 24 hour after last training (post-training). Data are mean±SD. *P<0.05 (compared with pre-training); #P<0.05 (NPPV vs non-NPPV).

At baseline, NPPV had no significant effects on $\dot{V}O_{2\max}$, HR_max_, VE_max_ and RR_max_ during training ($\dot{V}O_{2\max}$: no-NPPV: 52.5±4.7, NPPV: 53.0±4.3 ml/kg/min, HR_max_: no-NPPV: 189.1±6.1, NPPV: 188.4±5.6 bpm, VE_max_: no-NPPV: 145.9±21.1, NPPV: 153.1±32.6 l/min, RR_max_: no-NPPV: 59.7±5.2, NPPV: 58.4±7.9 breath/min). On the other hand, $\dot{V}O_{2\max}$ was significantly higher in post-training (under NPPV: 56.2±6.5 ml/kg/min, without NPPV: 54.0±6.4 ml/kg/min, P<0.05), compared with that at baseline (under NPPV: 53.0±4.3, without NPPV: 52.5±4.7 ml/kg/min). However, $\dot{V}O_{2\max}$ in post-training with NPPV (56.2±6.5 ml/kg/min) was significantly higher (P<0.05) than in post-training without NPPV (54.0±6.4 ml/kg/min) (Fig 3A). The delta change in $\dot{V}O_{2\max}$ during training under NPPV (3.2 ml/kg/min) was significantly higher (P<0.05) than during training without NPPV (1.5 ml/kg/min). HR_max_ and RR_max_ at pre- (HR_max_: under NPPV: 188.4±5.6, without NPPV: 189.1±6.1 bpm, RR_max_: under NPPV: 58.4, without NPPV: 59.7 breath/min) and post- (HR_max_; under NPPV: 188.9±5.0, without NPPV: 188.3±5.0 bpm, RR_max_; under NPPV: 62.9±7.2, without NPPV: 60.3±6.1 breath/min) training were not significantly different both with and without NPPV (Fig 3B and 3C). VE_max_ in post-training with NPPV (176.2±30.1 l/min), but not without NPPV (152.3±27.6 l/min), was significantly higher (P<0.05) than pre-training (under NPPV: 153.1±32.6, without NPPV: 145.9±21.1 l/min). Furthermore, VE_max_ in post-training with NPPV (176.2±30.1 l/min) was significantly higher (P<0.05) than in post-training without NPPV (152.3±27.6 l/min) (Fig 3D).

### Effects of NPPV on hemoglobin, hematocrit and plasma volume

**Fig 4:**
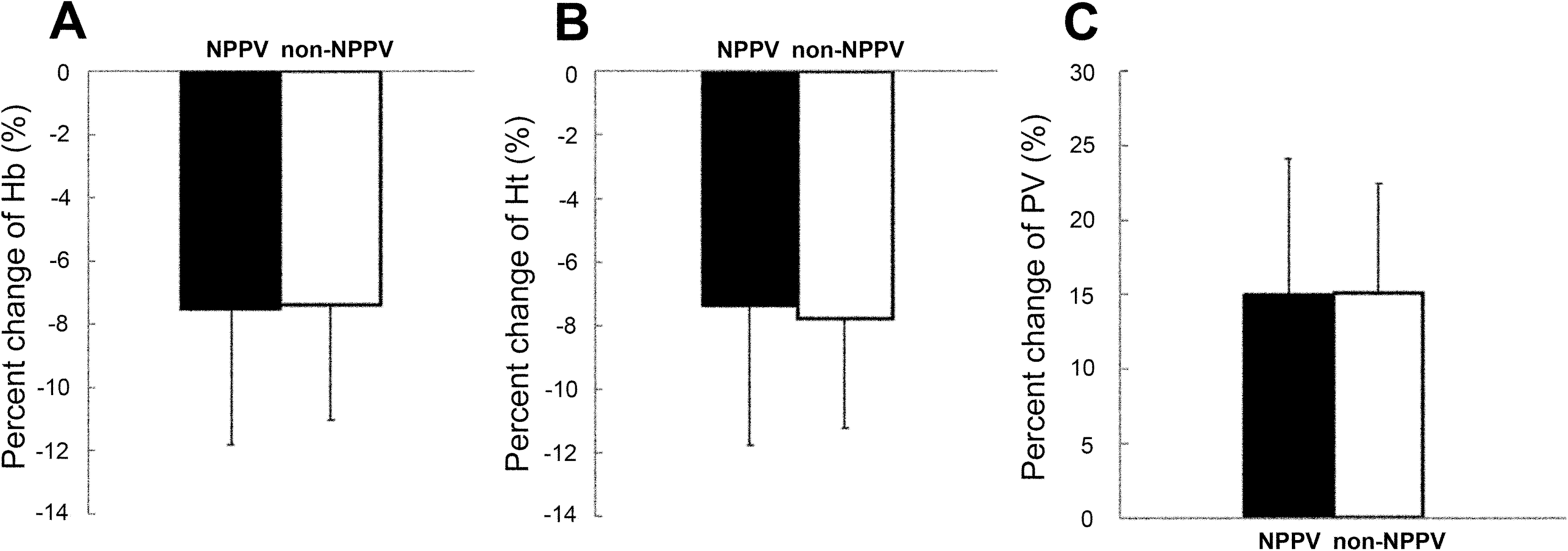
Percent changes in **(A)** hemoglobin (Hb), **(B)** hematocrit (Ht), and **(C)** plasma volume (PV) at 24 hour after last training (post-training) under NPPV and non-NPPV, compared with pre-training values. Data are mean±SD.

At baseline, hemoglobin concentration and hematocrit were not significantly different between with NPPV (15.5±0.8 g/dl, 45.7±1.9%, respectively) and without NPPV (15.2±1.1 g/dl, 45.1±3.0, respectively). Furthermore, the percent changes in hemoglobin concentration, hematocrit, and PV during training were not significantly different between with (-7.5±4.3%, -7.3±4.4%, 15.2±9.2%, respectively) and without NPPV (-7.4±3.7%, -7.8±3.4, 15.2±7.4%, respectively) (Fig 4A, 4B and 4C).

## Discussion

The followings are the major two findings of present study; *1)* 5-day aerobic training (ergometer exercise in an upright position at 60% of pre-training $\dot{V}O_{2\max}$ for 30 min/day) significantly increased $\dot{V}O_{2\max}$ with and without NPPV, but the magnitude of increase was significantly higher with 12 cmH_2_O NPPV than without NPPV in healthy young males. *2)* NPPV of 12 cmH_2_O significantly reduced SV and CO at rest. These findings suggest that NPPV at 12 cmH_2_O can reduce SV and CO, and that the same NPPV can further enhance the cardiopulmonary beneficial effects of aerobic training.

Positive pressure ventilation (PPV) with PEEP decreases SV and CO, but not BP and HR [23]. The major mechanism of SV and CO reduction is a decrease in venous return to the right heart secondary to increased intrathoracic pressure [24–26]. Our study also demonstrated that NPPV of 12 cmH_2_O reduced SV and CO at rest but did not change BP or HR.

$\dot{V}O_{2\max}$ is calculated by multiplying maximal cardiac output by maximal arterial-venous O_2_ difference ($\dot{V}O_{2\max}$ = CO_max_ × a-vO_2_ diff max) [9,27]. In addition, CO_max_ is also calculated by multiplying maximal SV (SV_max_) by HR_max_. Research suggests that vigorous aerobic training (60–84% $\dot{V}O_{2\max}$) results in a significant increase in cardiopulmonary function [28]. Moreover, vigorous aerobic exercise also increases SV by increasing blood volume and strength of cardiac contraction, leading to improvement in $\dot{V}O_{2\max}$ [9,29]. In the present study, 5-day vigorous aerobic exercise (cycle ergometer exercise at 60% of pre-training $\dot{V}O_{2\max}$ for 30 min/day) also significantly increased $\dot{V}O_{2\max}$ with and without NPPV. In addition, the same program increased PV, but not HR_max_. We assume that the increased $\dot{V}O_{2\max}$ after training with and without NPPV was probably induced by increases in blood volume and strength of cardiac contraction.

In the present study, the magnitude of increase in $\dot{V}O_{2\max}$ after the 5-day aerobic training at 12 cmH_2_O NPPV was significantly higher than that during the same length aerobic training without NPPV, though HR_max_ did not increase after either of the two protocols. As described above, $\dot{V}O_{2\max}$ is estimated by multiplying SV_max_ by HR_max_ and maximal arterial-venous O_2_ difference ($\dot{V}O_{2\max}$ = SV_max_ × HR_max_ × a-vO_2_ diff max). The results suggest that the increases in SV_max_ and/or a-vO_2_ diff max after training under NPPV could be larger than after training without NPPV.

The 5-day aerobic training significantly increased VE_max_ under NPPV only but not under the control condition. VE represents the product of tidal volume multiplied by respiratory frequency. Because the fastest respiratory rate is limited, any increase in VE_max_ is considered as a function of tidal volume, i.e., improvement in respiratory muscle contraction [30]. Resting ventilation is achieved by the contraction of inspiratory muscle activity with little or no expiratory muscle activity. During exercise, the associated hyperventilation involves increased inspiratory and expiratory muscle activities [31–33]. Respiratory muscle activity plays an important role in ventilatory control and plays an important role in respiratory response during exercise [34]. Previous studies reported that respiratory muscle training using respiratory resistance increased VE during exercise as well as respiratory muscle strength [35]. In the present study, the increase in VE_max_ after the 5-day aerobic training under NPPV could probably include NPPV-related increase in expiratory resistance. The increase in VE_max_ probably improved alveolar ventilation volume, and increased a-vO_2_ diff max. Therefore, the larger increase in $\dot{V}O_{2\max}$ after the 5-day aerobic training under NPPV compared with without NPPV could be related to improvement in alveolar ventilation volume and a-vO_2_ diff max.

Aerobic training increased PV. The latter contributes to the increase in SV_max_ and $\dot{V}O_{2\max}$ after aerobic training [9,29]. In the present study, the 5-day aerobic training also increased PV, and the percent increase in PV was not influenced by NPPV. Therefore, the larger increase of $\dot{V}O_{2\max}$ after 5-day aerobic training under NPPV compared with no NPPV is probably not due to changes in PV.

Several investigators reported that aerobic training without NPPV improves the strength of cardiac contraction and increases both SV_max_ and $\dot{V}O_{2\max}$ [9,29]. The pulmonary capillary-wedge pressure is similar to left ventricular end-diastolic pressure during 10 cmH_2_O PEEP or less, but left ventricular end-diastolic pressure was decreased by over 10 cmH_2_O PEEP [36]. The decrease in left ventricular end-diastolic pressure probably explains the reductions in SV and CO. In the present study, during aerobic exercise with 12 cmH_2_O NPPV, the absolute value of BP was maintained and end-diastolic pressure of the left ventricle should decrease. Thus, the total pressure production by the myocardia during exercise under NPPV should be greater than without NPPV. We assumed that the relative afterload in the myocardia would increase and the cardiac workload should be elevated during exercise with NPPV. Based on these suggestions, exercise under 12 cmH_2_O NPPV could increase cardiac stress, with resultant improvement in cardiac contraction. The larger increase in $\dot{V}O_{2\max}$ after 5-day aerobic training under NPPV (compared to under no NPPV) would be at least in part due to improvement in the strength of cardiac contraction.

Middle-aged and elderly people with cardiopulmonary dysfunction, low $\dot{V}O_{2\max}$ and obesity are prone to develop adult-related diseases, e.g., diabetes mellitus, cardiovascular disease and dyslipidemia [37,38]. Therefore, exercise improves $\dot{V}O_{2\max}$ and is important in preventing the development of adult diseases in middle-aged and elderly people. In the present study, aerobic exercise training under NPPV further improved $\dot{V}O_{2\max}$. Aerobic exercise training under NPPV could be beneficial clinically in preventing adult-related diseases. On the other hand, cardiopulmonary function is poor in individuals with physical disabilities, e.g., spinal cord injury, partly due to low physical activities of daily living [39]. Furthermore, cardiopulmonary function in astronauts during space flight is reduced due to microgravity, and prevention of such reduction is important in the field of space medicine [40]. We believe that aerobic exercise training under NPPV can prevent cardiopulmonary dysfunction in disabled people and astronauts.

The present study has certain limitations. We could not measure directly differences in SV_max_ between aerobic training under NPPV and no-NPPV due to technical difficulty. Because of this, we could not directly compare the difference in the present study. Moreover, the subjects included in the present study were healthy young men, and the results may not be applicable to children, women and elderly people. Further studies are needed to measure SV_max_ directly after overcoming these technical difficulties, and also to evaluate the response to different conditions of exercise stress and length, as well as the response in females and males of different age groups.

In conclusion, the present study examined the effects of aerobic exercise training under NPPV as a new endurance training method. The results showed that 5-day aerobic endurance training at 60% of pre-training VO_2max_ for 30 min/day under NPPV resulted in greater improvement of VO_2max_ than training without NPPV in healthy young men. The results suggest that aerobic exercise training under NPPV has an add-on effect on VO_2max_ and exercise-related health benefits in healthy young men.

## Supporting information

S1 File Raw data of the present study. (DOCX)
