# Supplementary material for: Noninvasive positive pressure ventilation enhances the effects of aerobic training on cardiopulmonary function
Source: PLoS One. 2017 May 22;12(5):e0178003. doi: 10.1371/journal.pone.0178003 (PMC5439726; doi:10.1371/journal.pone.0178003)
Supplement: S1 File — (DOCX) [file pone.0178003.s001.docx]

**Supporting Information**

S1 File. Raw data of the present study.

(XLSX)
